# Ancient DNA Elucidates the Controversy about the Flightless Island Hens (*Gallinula* sp.) of Tristan da Cunha

**DOI:** 10.1371/journal.pone.0001835

**Published:** 2008-03-19

**Authors:** Dick S. J. Groenenberg, Albert J. Beintema, René W. R. J. Dekker, Edmund Gittenberger

**Affiliations:** 1 National Museum of Natural History Naturalis, Leiden, The Netherlands; 2 Biowrite, Gorssel, The Netherlands; 3 Institute of Biology, Leiden University, Leiden, The Netherlands; Ecole Normale Supérieure de Lyon, France

## Abstract

A persistent controversy surrounds the flightless island hen of Tristan da Cunha, *Gallinula nesiotis*. Some believe that it became extinct by the end of the 19th century. Others suppose that it still inhabits Tristan. There is no consensus about *Gallinula comeri*, the name introduced for the flightless moorhen from the nearby island of Gough. On the basis of DNA sequencing of both recently collected and historical material, we conclude that *G. nesiotis* and *G. comeri* are different taxa, that *G. nesiotis* indeed became extinct, and that *G. comeri* now inhabits both islands. This study confirms that among gallinules seemingly radical adaptations (such as the loss of flight) can readily evolve in parallel on different islands, while conspicuous changes in other morphological characters fail to occur.

## Introduction

**Figure 1:**
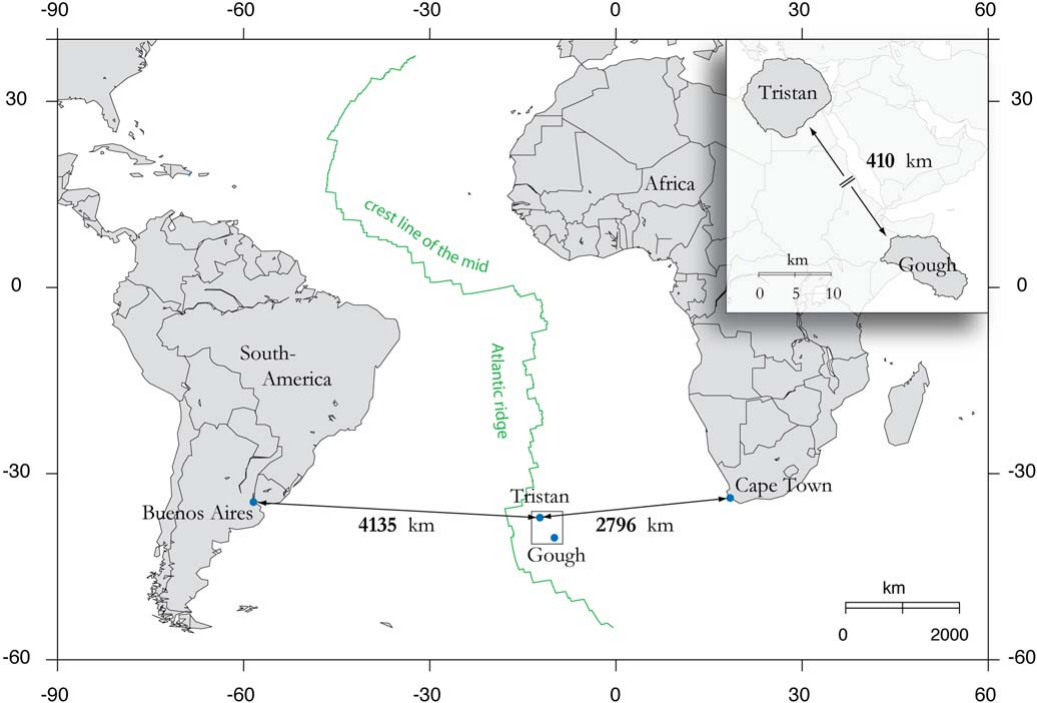
Map showing the location of Tristan da Cunha and Gough in the Mid Atlantic.

**Figure 2:**
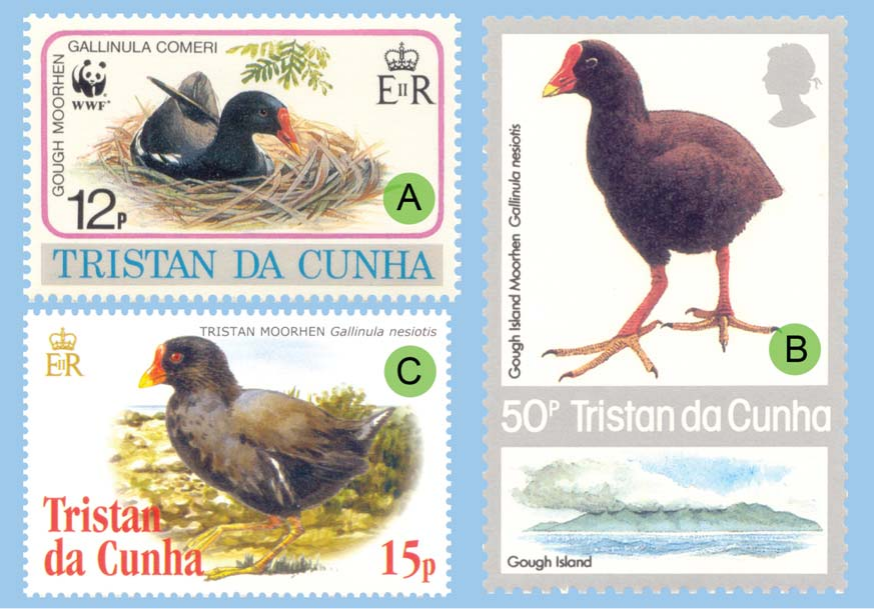
Illustrative stamps, issued in 1987 and 2005. (A) 2005: Text and illustrations belong together and are correct. (B) 1987: In Gough *G. comeri* occurs, not *G. nesiotis*; both names should not be synonymized. (C) 2005: The text correctly indicates *G. nesiotis* as from Tristan, but the bird itself most probably belongs to *G. comeri*, introduced from Gough, since *G. nesiotis* is now extinct on Tristan and not available to be pictured anymore.

Until recently it was assumed that the flightless moorhen of remote Tristan da Cunha in the southern Atlantic (Fig. 1), *Gallinula nesiotis* (Sclater, 1861) [1], became extinct by the end of the 19th century [2]. A few decades after its description, a very similar moorhen that was also flightless namely *G. comeri* (Allen, 1892), was described [3] from the island of Gough, ca. 400 km SE of Tristan. In the period between these descriptions *G. nesiotis* became rare [4], [5] and by the turn of the century it had probably gone extinct [7], [6]. Authentic remnants are two skins and a skeleton in the Natural History Museum, Tring [8]. Since unequivocal *G. nesiotis* had been collected only once from Tristan, and because of the presence of a healthy population of similar moorhens on the nearby island of Gough, some authors doubted whether an endemic moorhen had ever existed on Tristan [9]. Eber [10] compared Sclater's description of *G. nesiotis* from Tristan with her series of *G. comeri* from Gough and concluded that the differences fall within the range of variation of the latter. In her opinion it was very unlikely that moorhens from two islands in the same region would have independently lost the ability of flight, without differentiating in other characters. She suggested that Sclater's material might have been labelled inaccurately and that his specimens in fact also came from Gough. Consequently, Eber considered *G. comeri* a junior synonym of *G. nesiotis* [10] and controversy surrounded future illustrations of both taxa (Fig. 2).

Beintema [2] mentioned that there are old records of moorhens for Tristan, demonstrating that such birds were truly indigenous there. In his view, skeletal measurements differ slightly between *G. nesiotis* and *G. comeri*. Furthermore, rails are known to rapidly lose the ability of flight as soon as they arrive on remote islands [11]. When in 1972 live moorhens were discovered on Tristan [12], these birds were regarded as descendants of a small number of individuals brought from Gough [13]. Alternatively, Beintema suggested that *G. nesiotis* might have been temporarily rare on Tristan, but not extinct, and that the moorhens found there today are descendents of the original island population. Here we address this question, making use of DNA analyses of authentic material of *G. nesiotis*, recent specimens of the moorhens from both Tristan and Gough and some geographically and taxonomically close other taxa of moorhens.

## Results

An overview of the specimens that were used in this study, with taxon names, locality data, and year of acquisition, is given in Table 1. Alignments of all cloned sequences of *G. nesiotis* (two independent amplifications per target region) are shown in Dataset S1, S2, S3. On the one hand, none of the sequences of genuine, historical *G. nesiotis* was identical to those of *G. comeri* and, on the other hand, all sequences of the moorhens collected in Tristan da Cunha in 1993, are identical to those found for specimens of *G. comeri* from Gough, dated 1960. The genetic distances between *G. nesiotis* and the other gallinules, are of the same magnitude as the distances between *G. comeri* and the other gallinules (Table 2). A pairwise relative rates test did not reveal significantly different substitution rates for any of the lineages (P>0.18). Of the selected markers, most variation was detected in the control region (D-loop) sequences, viz. 9.6% TSH for *G. nesiotis* compared with *G. comeri* ( =  Total Sequence Heterogeneity [15], [14]) versus 2.1% and 0.3% TSH for tRNA-Lys/ATP synthase subunit 8 (ATP8) and cytochrome *b*, respectively.

**Figure 3:**
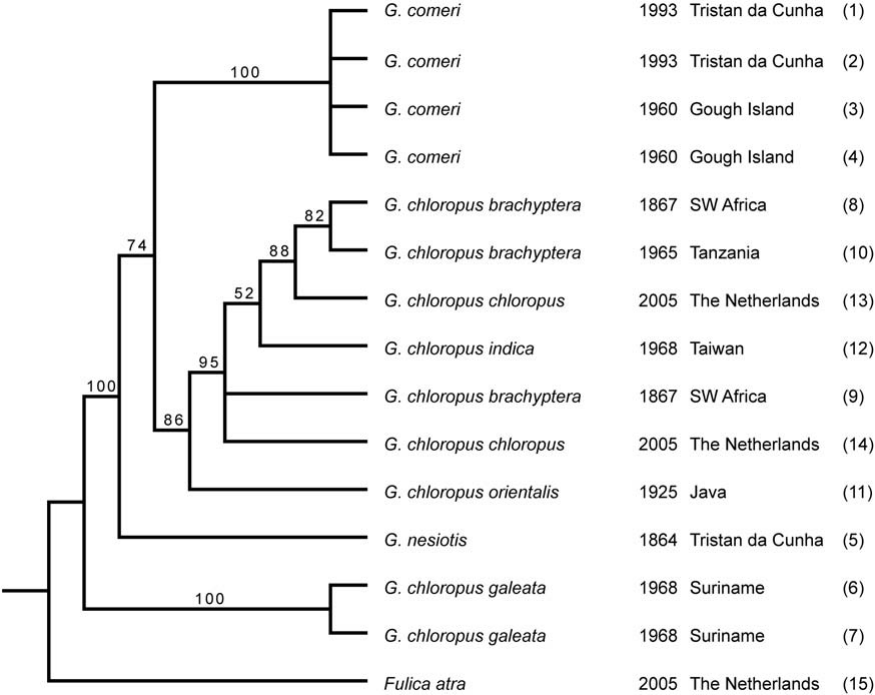
Bootstrap 50% majority rule consesus NJ tree. Values indicate bootstrap support.

The results of phylogenetic analyses (Neighbour-Joining, Maximum Likelihood and Bayes) based on a combined dataset (all taxa, all regions, Dataset S4) are shown in Fig. 3–5. In these cladograms *Gallinula nesiotis* and *G. comeri* form a clade with the moorhens of Africa/Eurasia, whereas the other taxa that were investigated are less closely related.

**Figure 4 pone-0001835-g004:**
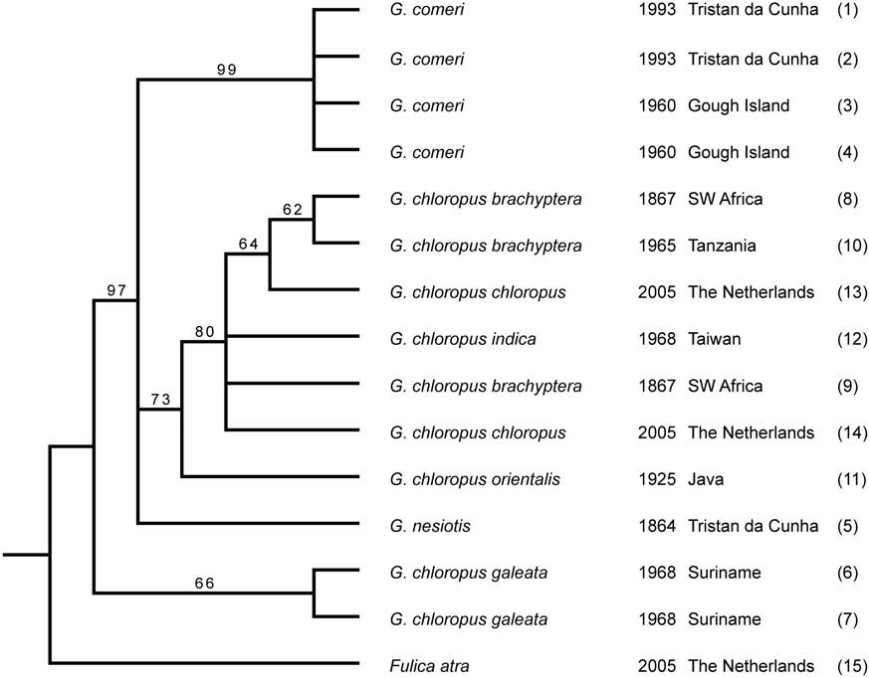
Bootstrap 50% majority rule consensus ML tree. Values indicate bootstrap support.

**Figure 5:**
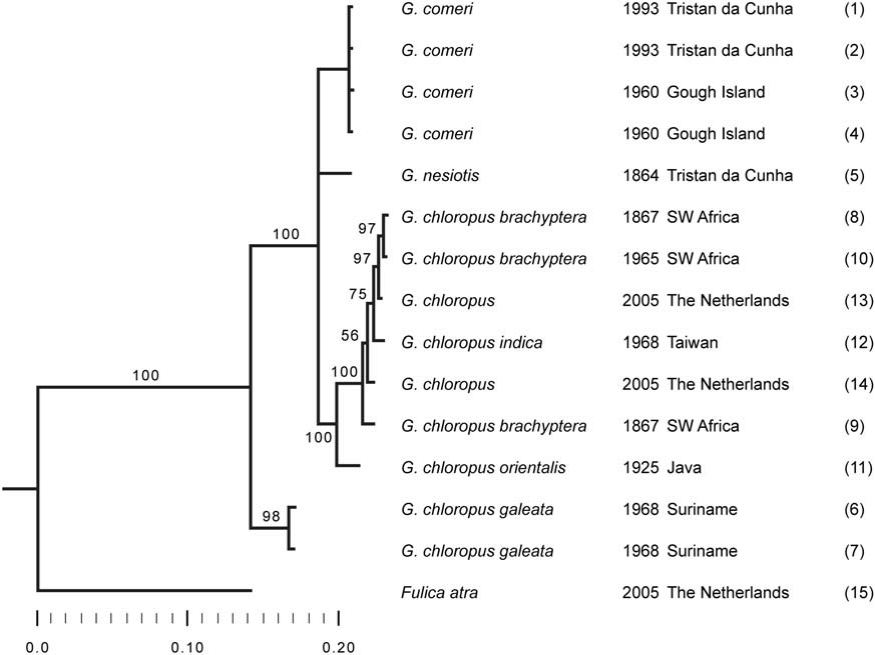
Bayes 50% majority rule consensus (‘halfcompat’) phylogram (branch lengths are proportional to the expected number of substitutions). Values indicate branch support by Bayesian inference.

**Table 1:** Taxa and collection information.

	Species	Locality	Registration number	Year	Institute
(1)	*G. comeri*	Tristan da Cunha	AB1759	1993	Pers. col. A.J. Beintema
(2)	*G. comeri*	Tristan da Cunha	AB1760	1993	Pers. col. A.J. Beintema
(3)	*G. comeri*	Gough Island	Cat. no 7 ZMA 14695	1960	ZMA*
(4)	*G. comeri*	Gough Island	Cat. no 14 ZMA 14696	1960	ZMA*
(5)	*G. nesiotis*	Tristan da Cunha	1864.7.30.1	1864	BM*
(6)	*G. chloropus galeata*	Suriname	RMNH 53835	1968	NNM*
(7)	*G. chloropus galeata*	Suriname	RMNH 53836	1968	NNM*
(8)	*G. chloropus brachyptera*	S.W. Africa	Cat. no. 21	1867	NNM*
(9)	*G. chloropus brachyptera*	S.W. Africa	Cat. no. 22	1867	NNM*
(10)	*G. chloropus brachyptera*	Tanzania	RMNH 43858	1965	NNM*
(11)	*G. chloropus orientalis*	Cheribon, Java	Cat. no 94 RMNH 26803	1925	NNM*
(12)	*G. chloropus indica*	Chang Hwa, Taiwan	Cat. no 12 RMNH 53054	1968	NNM*
(13)	*G. chloropus chloropus*	The Netherlands	DG2073	2005	NNM*
(14)	*G. chloropus chloropus*	The Netherlands	DG2077	2005	NNM*
(15)	*Fulica atra*	The Netherlands	DG2071	2005	NNM*

* ZMA = zoologisch museum Amsterdam, BM = Natural History Museum, Tring, NNM = nationaal natuurhistorisch museum, Naturalis.

**Table 2:** Genetic distances.

		*comeri* (1)	*comeri* (2)	*comeri* (3)	*comeri* (4)	*nesiotis* (5)
(1)	*G. comeri*					
(2)	*G. comeri*	0/0.000				
(3)	*G. comeri*	0/0.000	0/0.000			
(4)	*G. comeri*	0/0.000	0/0.000	0/0.000		
(5)	*G. nesiotis*	21/0.031	21/0.031	21/0.031	21/0.031	
(6)	*G. c. galeata*	40/0.059	40/0.059	40/0.059	40/0.059	41/0.061
(7)	*G. c. galeata*	41/0.060	41/0.060	41/0.060	41/0.060	40/0.059
(8)	*G. c. brachyptera*	20/0.035	20/0.035	20/0.035	20/0.035	24/0.042
(9)	*G. c. brachyptera*	22/0.038	22/0.038	22/0.038	22/0.038	24/0.042
(10)	*G. c. brachyptera*	22/0.032	22/0.032	22/0.032	22/0.032	25/0.037
(11)	*G. c. orientalis*	19/0.033	19/0.033	19/0.033	19/0.033	21/0.037
(12)	*G. c. indica*	21/0.031	21/0.031	21/0.031	21/0.031	23/0.034
(13)	*G. c. chloropus*	21/0.031	21/0.031	21/0.031	21/0.031	24/0.035
(14)	*G. c. chloropus*	22/0.032	22/0.032	22/0.032	22/0.032	25/0.037
(15)	*F. atra*	104/0.154	104/0.154	104/0.154	104/0.154	100/0.148

		*galeata* (6)	*galeata* (7)	*brachypt.* (8)	*brachyp.* (9)	*brachyp.* (10)
(6)	*G. c. galeata*					
(7)	*G. c. galeata*	3/0.004				
(8)	*G. c. brachyptera*	42/0.073	40/0.070			
(9)	*G. c. brachyptera*	44/0.076	42/0.073	6/0.010		
(10)	*G. c. brachyptera*	43/0.063	42/0.062	0/0.000	6/0.010	
(11)	*G. c. orientalis*	40/0.070	40/0.070	17/0.030	15/0.026	17/0.030
(12)	*G. c. indica*	46/0.068	45/0.066	5/0.009	7/0.012	5/0.007
(13)	*G. c. chloropus*	44/0.065	43/0.063	1/0.002	5/0.009	1/0.001
(14)	*G. c. chloropus*	44/0.065	43/0.063	4/0.007	4/0.007	4/0.006
(15)	*F. atra*	93/0.138	94/0.139	93/0.163	91/0.160	103/0.152

		*orientalis* (11)	*indica* (12)	*chlorop.* (13)	*chlorop.* (14)
(11)	*G. c. orientalis*				
(12)	*G. c. indica*	18/0.031			
(13)	*G. c. chloropus*	16/0.028	4/0.006		
(14)	*G. c. chloropus*	15/0.026	5/0.007	3/0.004	
(15)	*F. atra*	94/0.166	105/0.155	104/0.154	103/0.152

Cell values show: absolute number of changes/uncorrected “p” distances.

## Discussion

Our results show that genuine *G. nesiotis*, identified on the basis of historical material from the island of Tristan da Cunha, differs genetically from *G. comeri*, which has been described from the island of Gough. For each marker the sequence of *G. nesiotis* differs from that of *G. comeri*, as well as from all other *Gallinula* taxa that were analysed, but the amount of variation differs strongly between the regions studied. The position of *G. nesiotis* on the cladograms (Fig. 3–5) makes sense biologically. Apparently, *G. nesiotis* became extinct on Tristan and *G. comeri* from Gough was introduced there, resulting in the current situation with *G. comeri* occurring on both islands. This implies that modern illustrations of so-called *G. nesiotis* from Tristan (Fig. 2) probably show introduced *G. comeri* from Gough.

The difference between *G. nesiotis* and *G. comeri* is most conspicuous in the D-loop sequence. This is a marker from a non-coding region, which makes it more difficult to exclude it as a potential pseudogene [16]. In some cases preferential amplification of numt (nuclear mitochondrial insertion) sequences has been observed [17], [18], but in most ancient DNA studies only occasional co-amplification of numts has been reported [19], [20]. This is not surprising since (particularly) in ancient DNA samples, mitochondrial DNA will be in excess over nuclear DNA. Consequently the incidence of numts should be reduced in such samples. To minimize the chance of amplifying numts, we did not use blood as a source for DNA-extractions in our recently collected material [17], [21] (in the older specimens it was not possible anyway). Because ‘universal’ primers may also be particularly prone to amplification of numts [17], the primers for the D-loop were made ‘gallinule-specific’. They did not even work for the closely related coot, *Fulica atra*. Products that were sequenced directly (both strands) showed only one signal, whereas multiple signals can be expected if both the target product and a numt would have been amplified. Interclone variation was low (Dataset S1, S2, S3). Only one sequence (*G. nesiotis*, marker ATP8) out of 96 clones (Table 3) could clearly be identified as a numt (Dataset S2). No stop-codons or frame-shift mutations were observed for the coding-region datasets (ATP8 and cytochrome *b*). No obvious deviations in either substitution rate (pairwise relative rate test) or base composition, like a decrease in GC content [22], [23], were observed.

**Table 3:** Number of colonies sequenced per target region per taxon.

	Taxon	Year	D-loop	tRNA-Lysine/Atp8	cytochrome *b*
(1)	*G. comeri*	1993	-	-	-
(2)	*G. comeri*	1993	-	-	-
(3)	*G. comeri*	1960	4	-	-
(4)	*G. comeri*	1960	-	-	-
(5)	*G. nesiotis*	1864	8 (5/3)*	14 (7/7)*	23 (6/5-6/6)*
(6)	*G. c. galeata*	1968	3	-	-
(7)	*G. c. galeata*	1968	4	-	-
(8)	*G. c. brachyptera*	1867	-	-	-
(9)	*G. c. brachyptera*	1867	5	-	-
(10)	*G. c. brachyptera*	1965	3	-	-
(11)	*G. c. orientalis*	1925	5	4	7 (7-0)*
(12)	*G. c. indica*	1968	4	5	7 (5-2)*
(13)	*G. c. chloropus*	2005	-	-	-
(14)	*G. c. chloropus*	2005	-	-	-
(15)	*F. atra*	2005	-	-	-

* Within parentheses are the number of colonies sequenced for each PCR product.

All sequences of recently collected moorhens from Tristan were identical to those of *G. comeri* from Gough and should be considered conspecific therefore. Cross contamination is very unlikely, since specimens from Tristan and Gough were amplified in different PCR-batches and contamination was not detected in other, partly much older specimens. Most probably the sequences are identical because *G. comeri* was introduced only recently on Tristan. Genetic variation within island populations is generally small compared to mainland populations [24]–[26]. For example, the giant tortoises (*Aldabrachelys*) of Mahé (Seychelles) and Mauritius (Mascare islands) still have identical sequences compared to those of Aldabra, from where they were shipped since the 1820s or earlier [27]. *G. comeri* may have been introduced on Tristan somewhere in the mid 1950s [12]. Assuming a generation time of two years, as known for *Gallinula chloropus*, *G. comeri* would only have had about only 20 generations (40 years) to differentiate on Tristan.

Both the genetic distances (Table 2) and the fact that *G. nesiotis* and *G. comeri* form a clade with the investigated moorhens of Africa/Eurasia (Fig. 3–5), suggest that the ancestor(s) of these island gallinules originated from Africa and not America, as suggested by Eber [10]. Our data do not allow us to distinguish between a single dispersal event to the archipelago, followed by allopatric differentiation, or two separate introductions from the continent to both Tristan and Gough.

As is inevitably the case with isolated island populations, the question of whether *G. nesiotis* and *G. comeri* were reproductively isolated under natural circumstances cannot be answered. Our limited data from a small number of specimens and sequences of only the mitochondrial lineage are insufficient to demonstrate hybridisation. Even though island populations generally show lower genetic variation than related mainland populations [24], the genetic distances between *G. nesiotis* and *G. comeri* are of at least the same magnitude as those found between taxa that figure as subspecies of *G. chloropus* in the literature (Fig. 5, Table 2). Therefore, we propose that the extinct moorhen of Tristan and the moorhens that live on Gough and Tristan today be regarded as subspecies, viz. *G. n. nesiotis* and *G. n. comeri*, respectively. This is in conformity with two recent, general checklists of the birds of the world [28], [29] and a detailed monograph of the rails of the world [13], but is different from a morphological study by Eber [10] in which both taxa are considered synonyms.

## Materials and Methods

### Taxa

Tissues from fourteen gallinules and a coot were put at our disposal by various institutes (Table 1). These include tissues of (I) ‘recently’ collected moorhens from Tristan da Cunha, (II) moorhens from Gough from the collection ZMA, (III) the 1864 specimen of *G. nesiotis* from the Natural History Museum, Tring, and (IV) a number of subspecies of *Gallinula chloropus* from South-America, Africa, Europe, Taiwan and Java from the National Museum of Natural History Naturalis, Leiden. The coot, *Fulica atra*, was used as outgroup.

### DNA extraction

DNA extractions on specimens from 1968 (Table 1) and older were carried out in a dedicated aDNA facility (LAF, Leiden, the Netherlands), which is physically isolated from the main laboratories. Before extractions took place, the extraction room was cleaned with a 0.05% bleach solution and the extraction-cabinet was decontaminated by turning on the UV lights at least 1 hour prior to the start of the extractions. No more than four extractions were done at once and negative controls were included with each set of extractions. Pippetes were cleaned with bleach and subsequently decontaminated (together with the dispossables) by UV irradiation (UV linker). Tissues were cut into small pieces to enlarge the contact surface between tissue and buffer. Total genomic DNA was extracted with a DNeasy Tissue Kit (Qiagen) using a prolonged incubation (24 hours). Proteinase K was added twice, once at the start and after 6 hours of incubation. To concentrate the extract, elution volume was decreased to 40 µl. Extractions on recently collected specimens (1993–2005) were done in a common lab, also using a DNeasy Tissue Kit (Qiagen).

### PCR and sequencing

PCRs were never performed in the aDNA facility and amplicons were never stored in this building. PCRs on the extract of the 1864 specimen were duplicated in different laboratories that are physically separated from each other as well as from the aDNA facility. In none of these labs had ever been worked on any species of gallinule before. Fragments from three non-adjacent mitochondrial gene regions (679 basepairs in total; primersites excluded) were amplified by PCR: the D-loop, tRNA-Lysine/ATP8 and cytochrome *b*. The length of these fragments (primer sites included) was 234, 236 and 375 bp, respectively. Primer sequences and references are described in Table 4. For *G. nesiotis* and (most) specimens of 1960 and older, cytochrome *b* could not be amplified directly using primers L14841 and H15149 [30]. Presumably because the DNA within these specimens got too degraded over time. Therefore, internal primers were designed (Table 4) to amplify this fragment in two overlapping parts: L14841- Rev219 (219 bp) and Fwd141- H15149 (249 bp). The primers for the D-loop (CR-OUD-F and CR-OUD-R) are ‘gallinule-specific’. For the coot, *Fulica atra*, the same region had to be amplified with other primers: CR-175-F and 12S-29-R (Table 4). PCRs were done using a standard Taq DNA Polymerase kit (Qiagen). Reaction volume was 25 µl and PCR conditions were 0.4 µM of each primer, 0.2 mM dNTP's and 5 units of Taq DNA Polymerase. For amplification of the cytochrome *b* and ATP8 regions, the final concentration of MgCl_2_ was 2.5 mM. For amplification of the D-loop fragment no MgCl_2_ was added (1.5 mM was already in the Qiagen PCR-buffer). Thermocycling conditions were 3 min. at 94°C (initial denaturation), followed by 40 cycles (15 sec. at 94°C, 30 sec. at AT°C and 40 sec. at 72°C) and final extension 5 min. at 72°C. Where AT is the anealling temperature for each primerset; 50°C for both cytochrome *b* and ATP8, 55°C for the D-loop fragment and 57°C for reamplification of cloned products (see below).

**Table 4:** PCR and sequencing primers.

Primer name	Primer sequence (5′ to 3′)	Target	Reference
L14841	AAAAAGCTTCCATCCAACATCTCAGCATGATGAAA	cytochrome *b*	Kocher, 1989
H15149	AAACTGCAGCCCCTCAGAATGATATTTGCCTCA	cytochrome *b*	Kocher, 1989
Fwd141	CCACACATGCCGCAACGTACAATA	cytochrome *b*	This study
Rev219	GCAGATGAAGAAGAATGAGGCTCC	cytochrome *b*	This study
L9051	CAGCACTAGCCTTTTAAG	tRNA-Lys/ATP8	Slikas, 2002
H9241	TTGGTCGAAGAAGCTTAGGTTCA	tRNA-Lys/ATP8	Slikas, 2002
CR-OUD-F	CCAAGTGTTAATAGTATATGAGCTTACTCC	D-loop	This study
CR-OUD-R	TGATACATTTTGATTGTTTGGTATGAA	D-loop	This study
CR-175-F	GAGCATACTATTGGTTGACGTGAG	D-loop	This study
12S-29-R	TTTACACTGGAGTGCGGATACTTGCAT	D-loop	This study
21M13_F	TGTAAAACGACGGCCAGT	pCR 2.1-TOPO M13 priming site	TOPO TA Cloning kit
21M13_R	CAGGAAACAGCTATGACC	pCR 2.1-TOPO M13 priming site	TOPO TA Cloning kit

All PCR products from *G. nesiotis* and a number of PCR product from the D-loop of selected taxa (Table 3) were cloned using either pGEM®-T Easy Vector Sytems from Promega or Topo TA Cloning® from Invitrogen. At least three colonies were picked per plate and used to initiate reamplifications with primers 21M13_F and 21M13_R (Table 4). Reamplified products were cleaned using a Nucleospin® kit (Macherey-Nagel). Subsequently these products were sequenced either in-house on a Megabace™ 1000 DNA Analysis System (Amersham), or on a 3730xl DNA analyzer (Applied Biosystems) at Macrogen Inc. (Korea) using only primer 21M13_F. All other PCR products were cleaned (same procedure) and sequenced directly (both directions) with their respective PCR primers (Table 4). A summary of the specimens and the number of colonies sequenced per target region is given in Table 3. Sequences were assembled using Sequencher version 4.2 (Gene Codes Corporation) and aligned manualy using MacClade version 4.08 [31]. The sequences were deposited in GenBank (accession numbers EF681971-EF682015).

### Phylogenetic analysis

For phylogenetic analyses, all sequences (all regions; consensus sequences when products were cloned) were put in a single datamatrix (Dataset S4; an ILD-test showed no incongruence between the regions, p = 0.971) and *Fulica atra* was designated as outgroup. To get branch support values, we performed phylogeneticanalyses with three methods: Neighbour-Joining (PAUP ver. 4.0b2a [32]), Maximum Likelihood (PAUP ver. 4.0b2a [32]) and Bayesian analysis (Mr.Bayes ver. 3.1.2 [33]). For the NJ analysis, we performed a bootstrap analysis (1000 replicates, optimality criterion set to distance) and calculated a 50% majority rule consensus cladogram (Fig. 3). For the ML analysis, the HKY+G model was selected by Modeltest ver. 3.7 [34] with the following parameters: Tratio = 10.660, gamma shape parameter = 0.0941, base frequencies A = 0.3473, C = 0.3004, G = 0.1341, T = 0,2182 and proportion of invariable sites (pinvar) = 0. A bootstrap analysis (1000 replicates, 5 random additions per bootstrap replicate and TBR branch swapping) was performed and a 50% majority rule consensus tree was calculated (Fig. 4). For the MrBayes analysis, the best-fit model for each partition (four partitions: tRNA-Lysine and ATP8 were considered as two partitions) was selected by hLRT in MrModeltest ver. 2.2 [35]: D-loop (HKY+Y), tRNA-lysine (HKY), ATP8 (GTR+I) and cytochrome *b* (GTR+I). A dirichlet (1,1,1,1) prior was specified on the state frequencies for all partitions, except for the tRNA-Lysine partition, where the frequencies were equal. All partitions had different rates for transition and transversions (nst = 2), except for tRNA-Lysine (nst = 1). Among-site rate variation was equal for tRNA-Lysine, gamma-distributed for both D-loop and ATP8 and for cytochrome *b* a proportion of the sites was invariant. Two runs (set up for 10 000 000 generations) were performed simultaneously (4 chains per run) in MrBayes ver. 3.1.2 [34] and the convergence diagnostic was set to 0.009. A Markov chain Monte Carlo (MCMC) analysis was done with swapfreq = 2, temp = 0.002 and samplefreq = 100; convergence was reached after 165 000 generations. The trees of both runs (3302 in total) were combined (2702: burnin was set to 300) and a 50% majoritiy rule consensus tree (contype = halfcompat) was calculated (Fig. 5).

### Genetic distances

Genetic distances (absolute number of changes and uncorrected “p” distances) were calculated with Paup ver. 4.0b2a [32] based on the combined dataset (Dataset S4).

### Pairwise relative rates test

With *Fulica atra* specified as outgroup, a Pairwise Relative Rate Test [36] as implemented in HyPhy [37] using the HKY model (as specified by Modeltest ver. 3.7 [34]) was performed on the combined dataset (Dataset S4).

## Supporting Information

Dataset S1 Cloning results of *G. nesiotis* for ATP8.
(0.00 MB TXT)

Dataset S2 Cloning results of *G. nesiotis* for D-loop.
(0.00 MB TXT)

Dataset S3 Cloning results of *G. nesiotis* for cytochrome b.
(0.01 MB TXT)

Dataset S4 Combined dataset showing all taxa and all markers (D-loop, ATP8 and cytochrome b) used in this study.
(0.01 MB TXT)
